# Supplementary material for: Unusual mode of dimerization of retinitis pigmentosa-associated F220C rhodopsin
Source: Sci Rep. 2021 May 18;11:10536. doi: 10.1038/s41598-021-90039-3 (PMC8131606; doi:10.1038/s41598-021-90039-3)
Supplement: Supplementary file 1 — Supplementary Information. [file 41598_2021_90039_MOESM1_ESM.pdf]

SUPPORTING INFORMATION

Unusual mode of dimerization of retinitis pigmentosa-associated F220C rhodopsin

George Khelashvili[1,2,¶], Anoop Narayana Pillai[3*], Joon Lee[3*], Kalpana Pandey[3*], Alexander M. Payne[4], Zarek Siegel[5], Michel A. Cuendet[1,6], Tylor R. Lewis[7], Vadim Y. Arshavsky[7], Johannes Broichhagen[8], Joshua Levitz[3,¶], Anant K. Menon[3,¶]

[1]Department of Physiology and Biophysics, Weill Cornell Medical College, New York, NY, 10065
[2]Institute of Computational Biomedicine, Weill Cornell Medical College, New York, NY 10065
[3]Department of Biochemistry, Weill Cornell Medical College, New York, NY, 10065
[4]Tri-Institutional PhD Program in Chemical Biology, Weill Cornell Medical College, New York, NY, 10065
[5]Neurosciences Graduate Program, University of California San Diego, La Jolla, CA 92093
[6]Ludwig Institute for Cancer Research, University of Lausanne, and Department of Oncology, University Hospital of Lausanne, 1009, Lausanne, Switzerland; Swiss Institute of Bioinformatics, 1015 Lausanne, Switzerland
[7]Department of Ophthalmology, Duke University Medical Center, Durham, NC, 27710
[8]Leibniz-Forschungsinstitut für Molekulare Pharmakologie, Robert-Rössle-Str. 10, 13125 Berlin, Germany

[*]A.N.P., J. Lee and K.P. contributed equally to the experimental work

[¶]To whom correspondence should be addressed: George Khelashvili, gek2009@med.cornell.edu, Joshua Levitz, jtl2003@med.cornell.edu, and Anant K. Menon, akm2003@med.cornell.edu

Running title: Rhodopsin dimerization

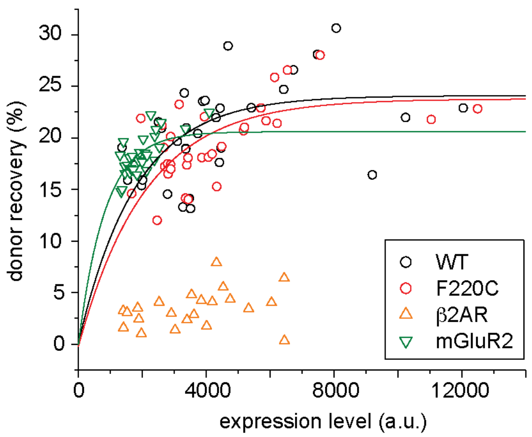

Figure S1. FRET measurements of N-terminally SNAP-tagged WT opsin, F220C opsin, β2-adrenergic receptor (β2AR) and metabotropic glutamate receptor 2 (mGluR2) expressed at the surface of HEK293T cells. Cells were transfected and taken for analysis after 24 and 36 h. Donor and acceptor dyes (SBG-JF549 and SBG-JF646, respectively) were used in a 1:3 ratio for labeling. FRET, reported as the percentage of donor fluorescence recovery after specific photobleaching of the acceptor population, is graphed against the expression level of the individual constructs. Donor bleaching correction was done by compensating 10% bleaching of donor (based on donor-only controls). Exponential curve fit was done in the OriginPro program using the formula y = y0 + A*Exp(x/t).

A

WT MONOMER

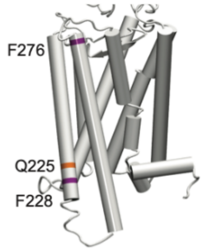

E

WT MONOMER

Residue	RHM (k_BT)
Q225	1.3 ± 0.4
F228	3.4 ± 0.4
F276	1.7 ± 0.3

B

F220C MONOMER

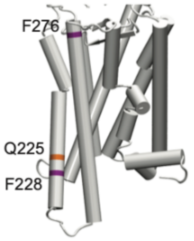

F

F220C MONOMER

Residue	RHM (k_BT)
Q225	1.1 ± 0.2
F228	3.3 ± 0.2
F276	1.8 ± 0.3

C

WT DIMER

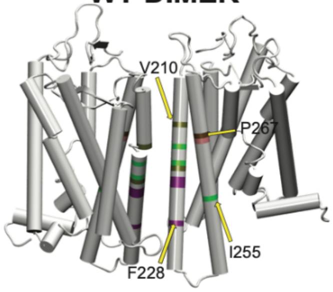

G

WT DIMER

Residue	RHM, Prot A (k_BT)	RHM, Prot B (k_BT)
V210	2.1 ± 0.7	2.1 ± 1.0
I214	2.1 ± 0.8	2.3 ± 0.8
I217	0.8 ± 0.4	1.3 ± 0.7
V218	1.5 ± 0.4	1.3 ± 0.4
F220	2.8 ± 1.1	2.1 ± 1.4
F221	1.4 ± 0.4	1.8 ± 0.3
F228	2.8 ± 0.9	0.7 ± 0.5
I255	1.1 ± 0.8	1.3 ± 1.2
L266	2.3 ± 1.2	1.5 ± 1.0
P267	1.1 ± 0.5	0.6 ± 0.6

D

F220C DIMER

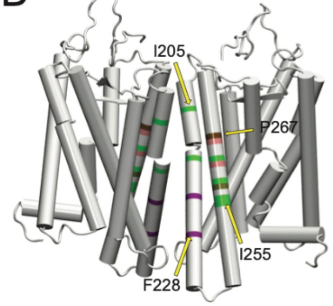

H

F220C DIMER

Residue	RHM, Prot A (k_BT)	RHM, Prot B (k_BT)
I205	1.0 ± 0.6	1.3 ± 0.7
I214	1.4 ± 1.2	1.6 ± 0.6
F221	1.2 ± 0.5	1.0 ± 0.6
F228	1.3 ± 0.8	2.6 ± 0.4
I255	2.4 ± 1.4	1.5 ± 0.7
I256	1.2 ± 0.8	0.3 ± 0.1
V258	1.1 ± 0.4	0.8 ± 0.4
I259	1.7 ± 1.3	0.3 ± 0.1
L262	1.2 ± 0.5	0.5 ± 0.3
I263	2.7 ± 1.3	0.3 ± 0.1
L266	1.7 ± 1.0	0.5 ± 0.2
P267	2.1 ± 0.3	0.3 ± 0.2

Figure S2: (**A-D**) Snapshots of WT and F220C monomer (A-B) and dimer (C-D) opsin constructs highlighting residues in TM5 and TM6 helices with high RHM penalty values. The residues are color coded based on their amino acid identity as following: Q – yellow, F – purple, I – green, L – pink, V – light brown, P – dark brown. Selected residues are labeled and marked with labels. (**E-H**) The RHM values for the residues highlighted in panels A-D. For the dimer systems, the RHM energies are separately shown for the two subunits (Prot A, Prot B). Color code of the residues is the same as in panels A-D.

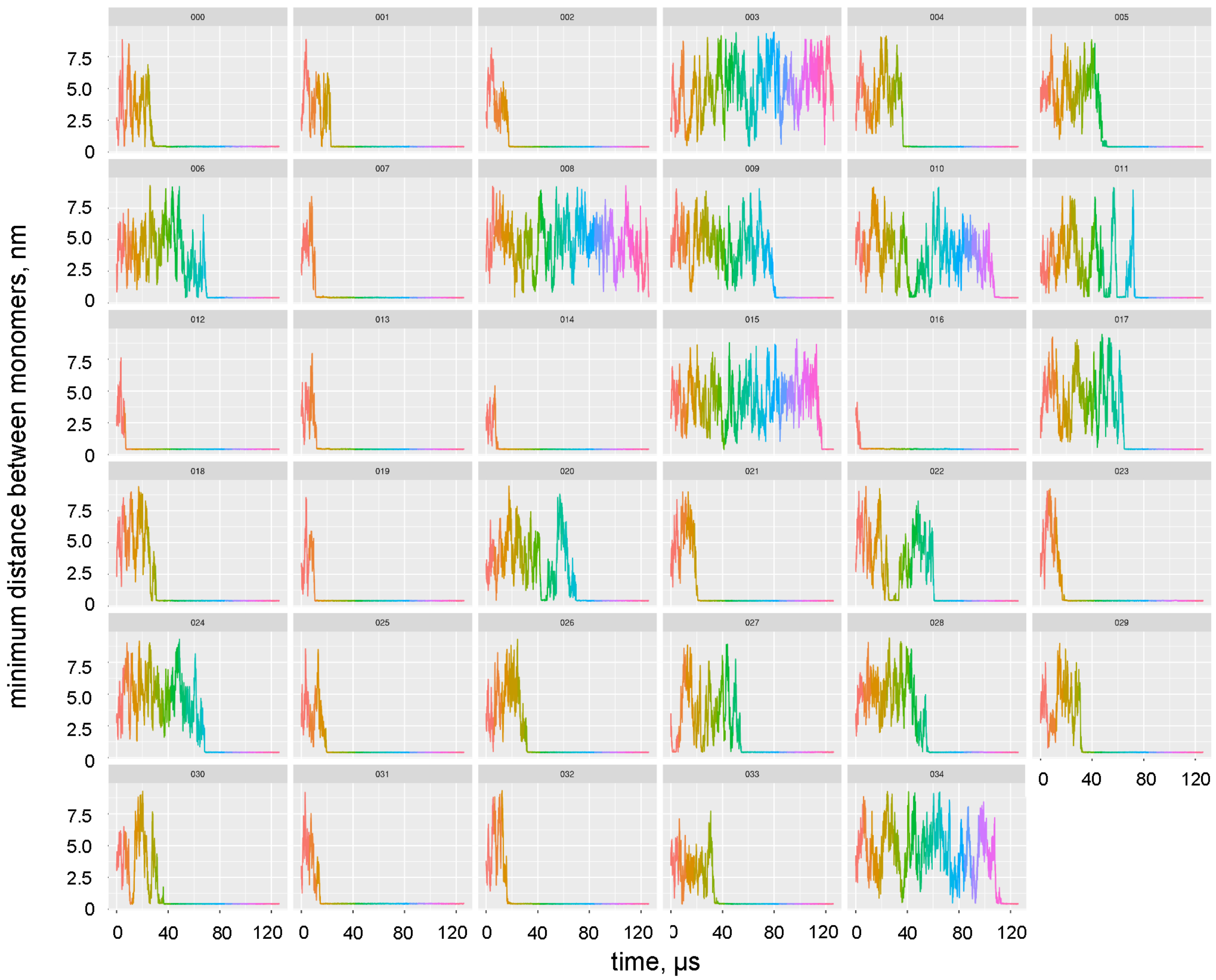

Figure S3: Time evolution of the minimum distance between two monomers of the WT opsin from the CG MD simulations. The data for each of the 35 replicates is shown in separate panels (labeled from 000-034). Small values of the distance reflect dimer formation. The rainbow color code in the panels, for clarity, represents simulation time.

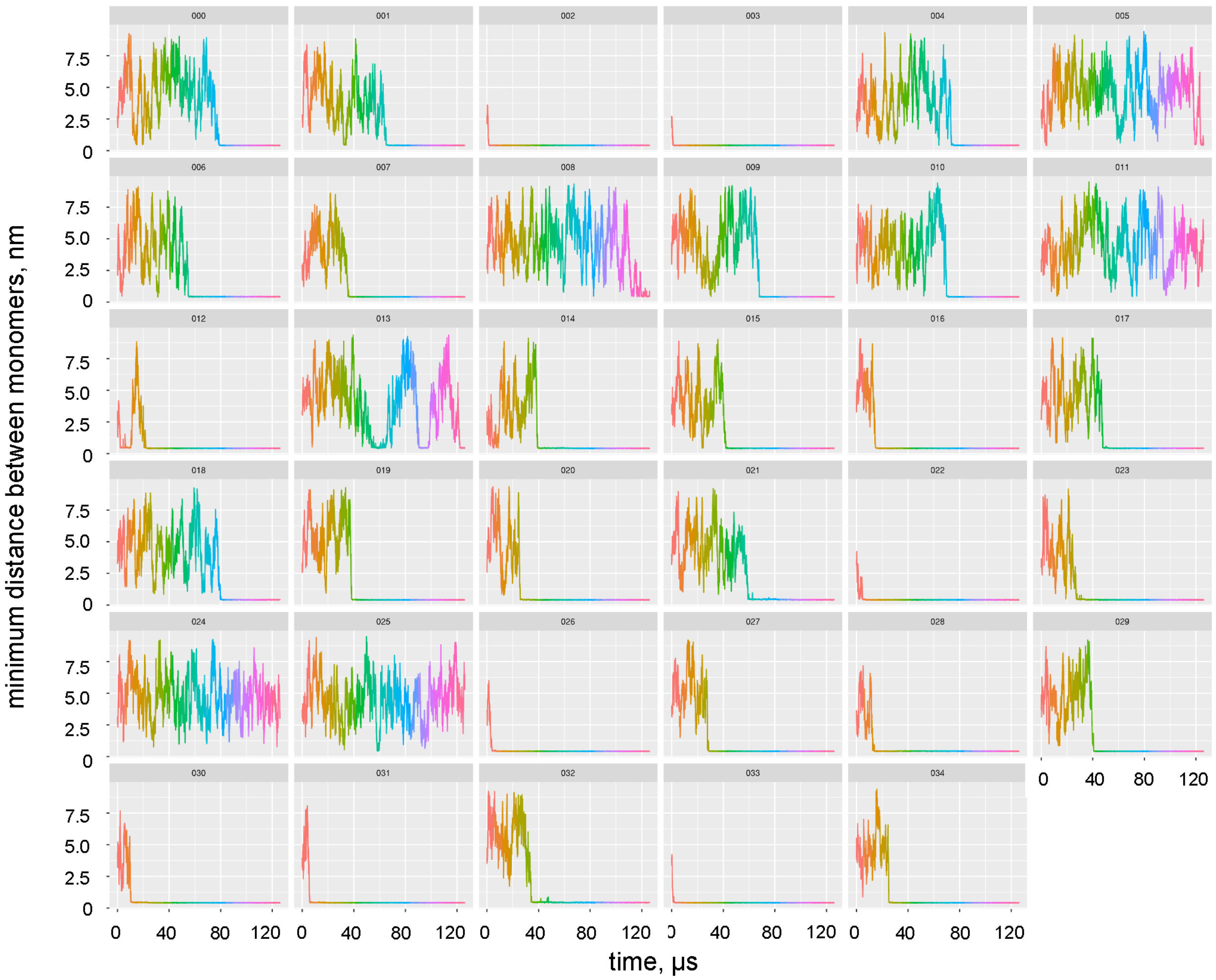

Figure S4: Time evolution of the minimum distance between two monomers of the F220C opsin from the CG MD simulations. The data for each of the 35 replicates is shown in separate panels (labeled from 000-034). Small values of the distance reflect dimer formation. The rainbow color code in the panels, for clarity, represents simulation time.

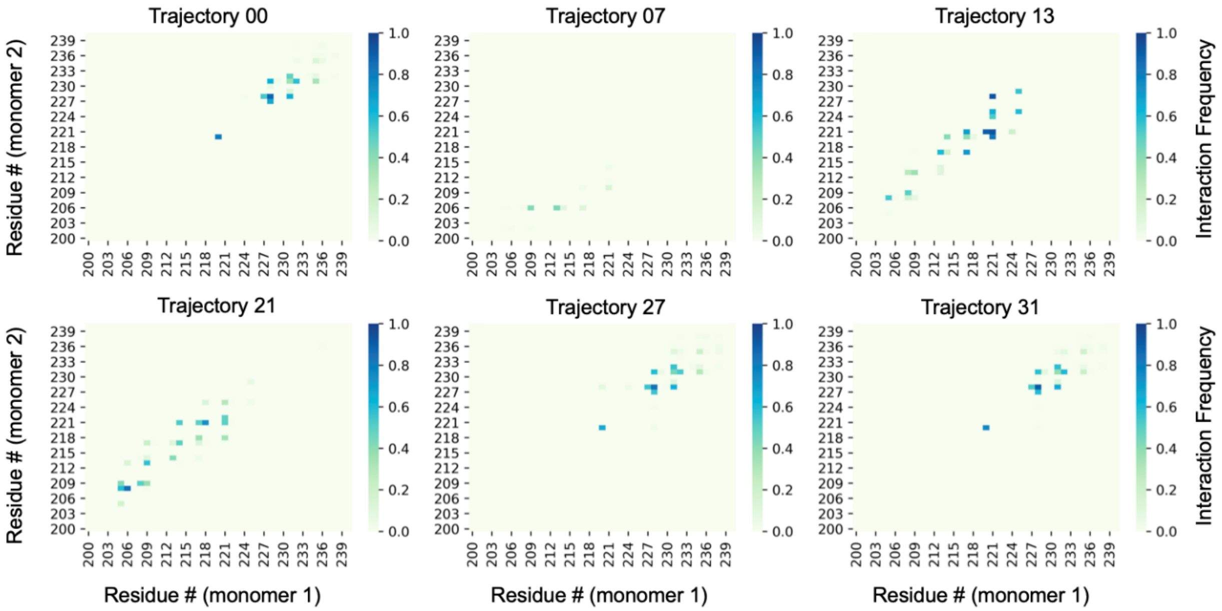

Figure S5: Pairwise residue contacts from the CG MD simulations of the WT opsin in which a dimerization interface was formed by interactions between TM5 segments on the opposite monomers of the dimer. The trajectory IDs are given on top of the panel. Color code represents normalized frequency of contacts between pairs of residues (see Methods for more details).

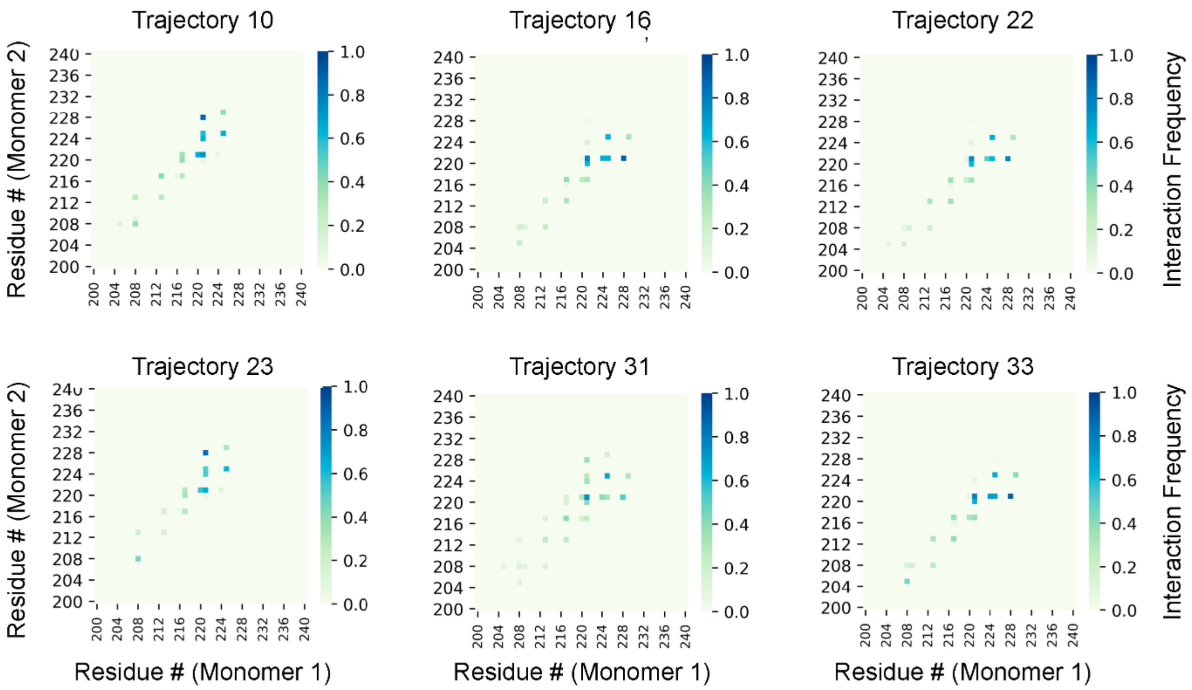

Figure S6: Pairwise residue contacts from the CG MD simulations of the F220C opsin in which a dimerization interface was formed by interactions between TM5 segments on the opposite monomers of the dimer. The trajectory ID-s are given on top of the panel. Color code represents normalized frequency of contacts between pairs of residues (see Methods for more details).

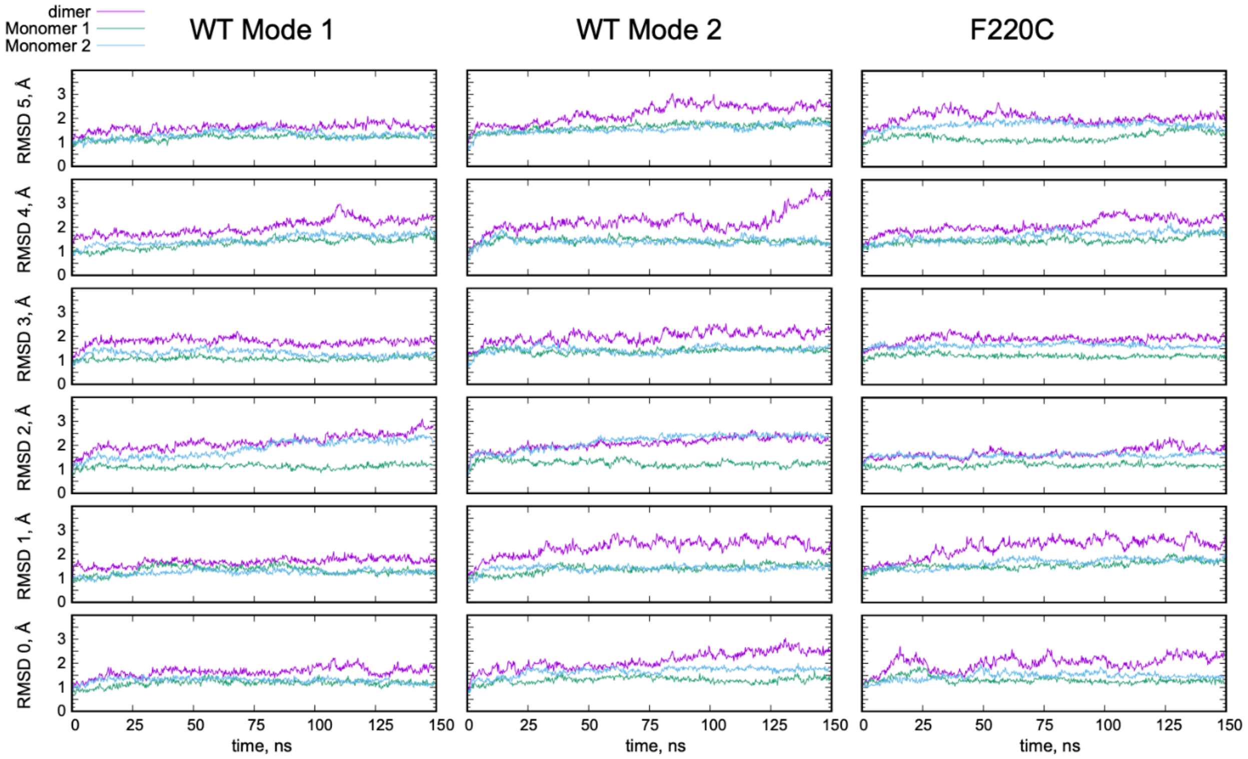

Figure S7: Time-evolution of root-mean-square deviation (RMSD) of the backbone atoms of the 7 transmembrane helical segments in the all-atom MD simulations of the Mode 1 and Mode 2 dimer models of the wild type opsin (WT Mode 1 and WT Mode 2), and of the F220C opsin dimer structure (F220C). The data for 6 independent replicates for each construct is given in separate rows. The RMSD was calculated for the entire dimer (purple traces), or for separate monomers (green and cyan traces). For each trajectory, the initial frame of the system was used as a reference structure for the RMSD calculations.

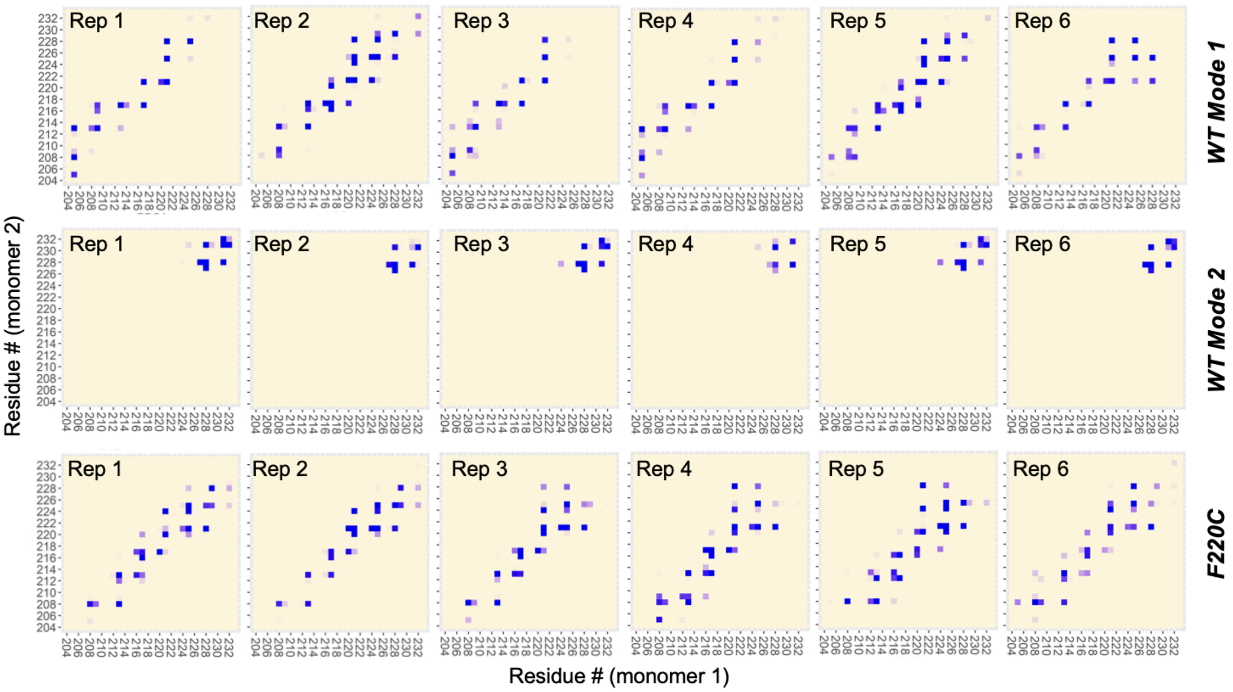

Figure S8: Pairwise residue contacts in the all-atom MD simulations of the two dimer models of the WT opsin (WT Mode 1 and WT Mode 2, top and middle rows) and of the F220C opsin dimer model (bottom row). The most frequent contacts are designated by dark blue shades, whereas the least frequent interactions are shown in light shades. The data for six independent replicates per construct is shown in separate panels, and the analysis was performed on the last 30ns of the all-atom trajectories.

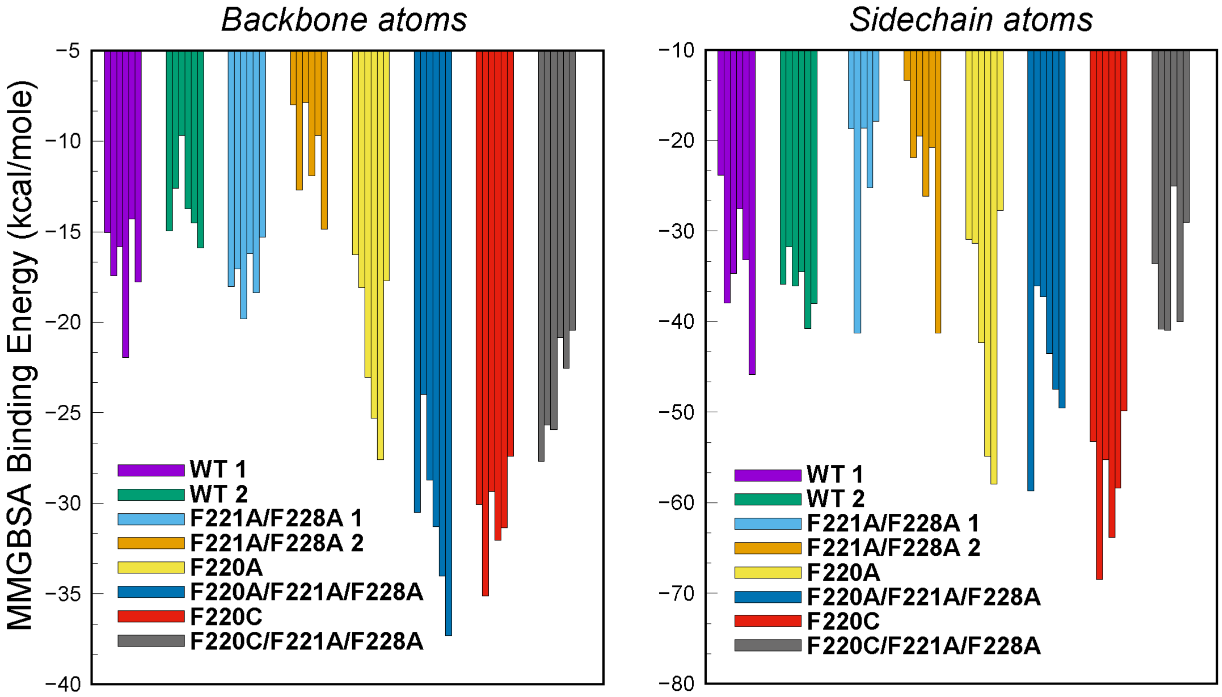

Figure S9: Contributions of the backbone (*left*) and sidechain (*right*) atom interactions to the overall MM-GBSA binding energy calculated from the all-atom MD simulations of the dimer models described in the text.

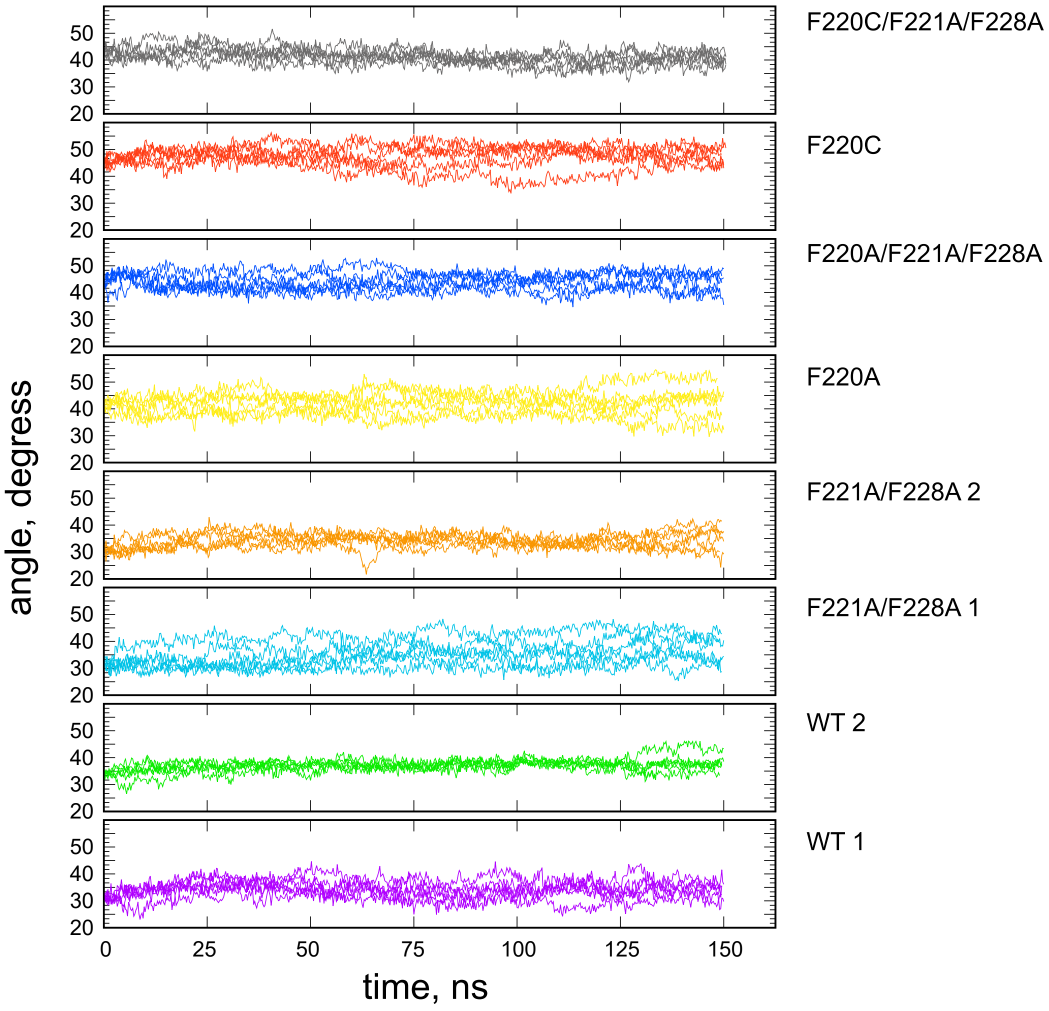

Figure S10: Time-evolution of the splay angle α in the all-atom MD simulations of the dimer models described in the text (see also Figure 7C in the main text). The color coding of the systems follows the same pattern as in Figures 6 and 7C in the main text. The results for each replicate are shown separately (i.e. 6 plots per constructs).

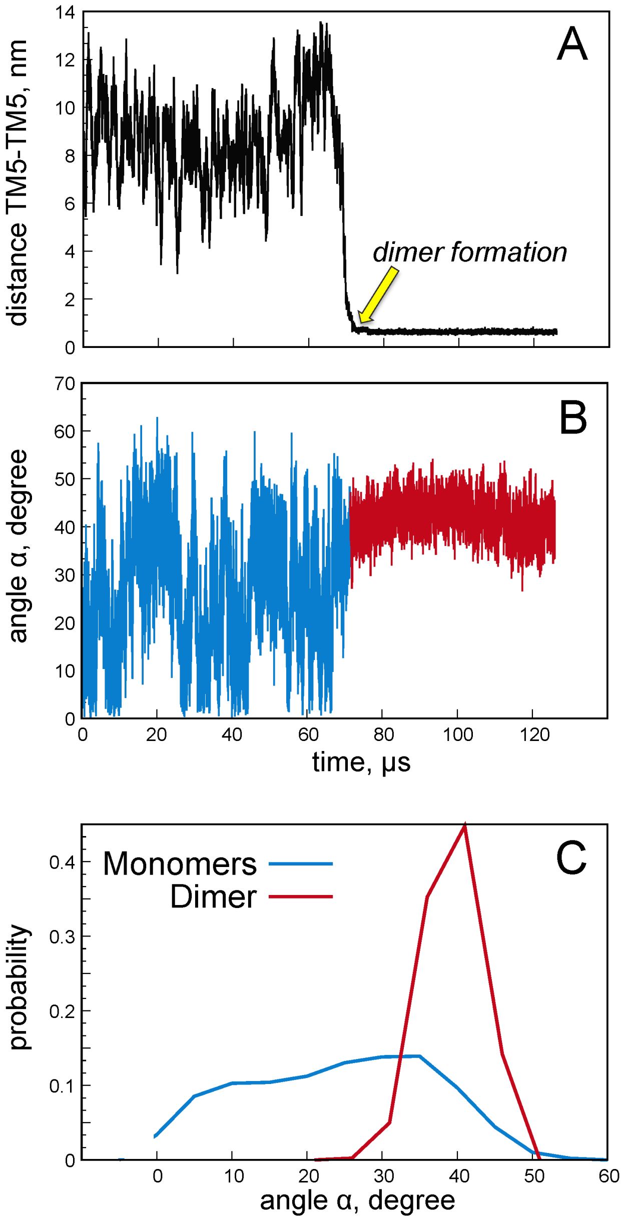

Figure S11: (A-B) Time evolution of the minimum distance between TM5 helices of the two monomers of opsin (A) and of the angle α between the two TM5 helices (B) in one of the CG MD simulations (Trajectory ID 10, see Figure S5). The time point of dimer formation is indicated with the yellow arrow. In panel B, the data for the trajectory parts before and after dimer formation are shown in blue and red colors, respectively. For definition of α angle see text. (**C**) Histograms of the α angle from panel B with the same color coding.

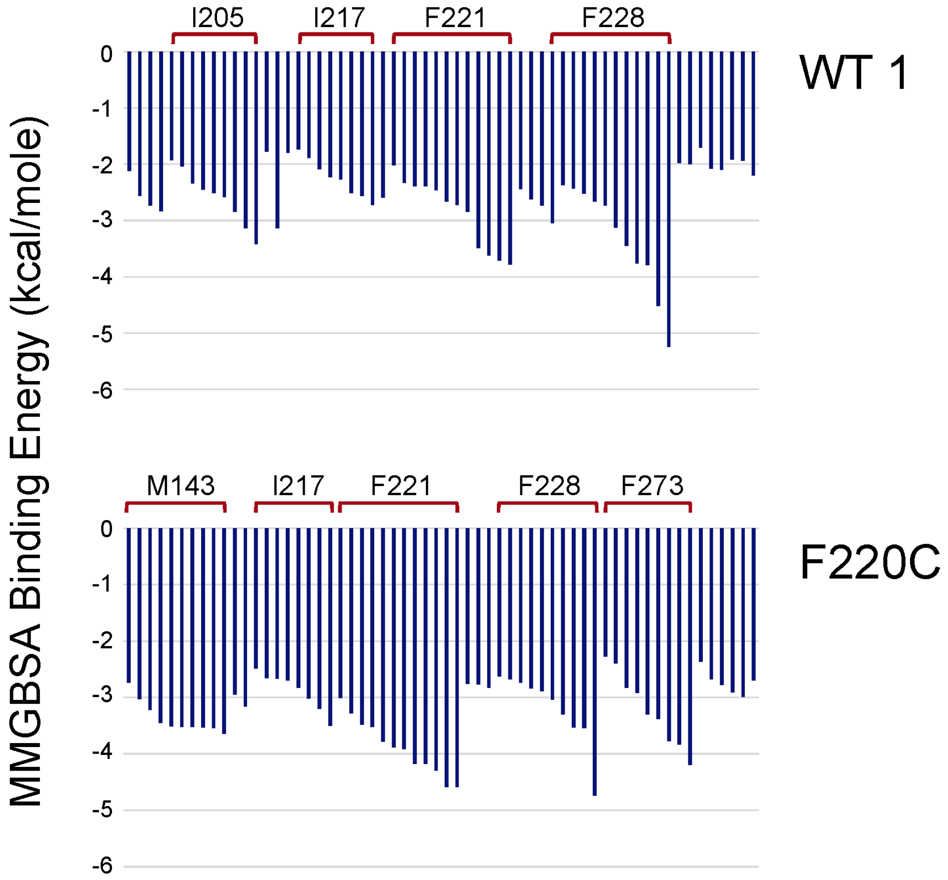

Figure S12: Per-residue decomposition of the MMGBSA binding energies for the WT Mode 1 and F220C dimer systems (upper and lower panels, respectively). Shown are contributions to the MMGBSA energy of the top 10 residue side-chains in all the trajectories for these two dimer constructs. The data is sorted according to residue ID from left to right. The data for the residues whose side-chains contributions were among top 10 in at least 4/6 trajectories are marked by red brackets and labeled.

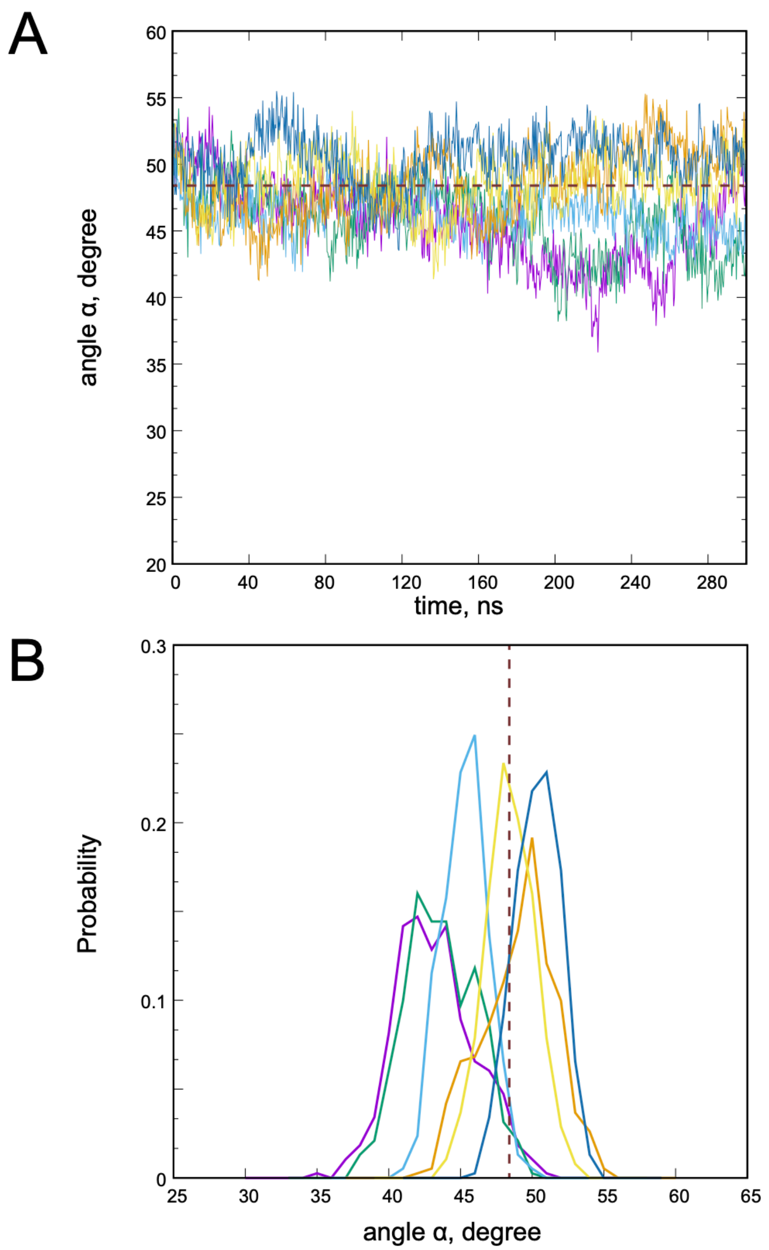

Figure S13: (**A**) Time-evolution of the α angle in the simulations of C220F opsin dimer. The data for 6 independent replicates are shown in different colors. The horizontal dotted line represents the value of the α angle (~48°) in the initial state of the system. (**B**) Histograms of the α angle from the panel A. The color scheme between the two panels is identical. The histograms were constructed from the analysis of the second halves of the MD trajectories described in panel A.

Prot. A /B	TM1	TM2	TM3	TM4	TM5	TM6	TM7	H8
TM1	6	6	0	0	3	2	1	1
TM2	7	4	1	0	2	3	0	0
TM3	7	4	0	0	1	0	0	1
TM4	9	9	0	0	0	0	0	3
TM5	8	4	2	1	6	5	0	0
TM6	2	2	0	2	6	5	0	1
TM7	2	3	1	1	0	0	0	0
H8	1	0	0	0	3	2	0	4

Table S1: Number of CG MD trajectories (out of 35 total replicates) of the WT opsin in which the dimerization was established via interactions between a specific pair of helical regions of the protein. 'H8' denotes helix 8 segment.

Prot. A /B	TM1	TM2	TM3	TM4	TM5	TM6	TM7	H8
TM1	8	8	3	2	5	3	0	2
TM2	7	3	1	1	4	2	0	0
TM3	4	1	0	0	6	0	0	2
TM4	1	1	0	0	0	1	0	0
TM5	4	3	6	0	7	5	0	3
TM6	2	5	0	0	6	6	0	1
TM7	1	1	0	0	0	0	1	0
H8	3	0	1	2	3	0	0	6

Table S2: Number of CG MD trajectories (out of 35 total replicates) of the F220C opsin in which the dimerization was established via interactions between a specific pair of helical regions of the protein. 'H8' denotes helix 8 segment.

	WT 1	WT 2	2F/A 1	2F/A 2	F220A	3F/A	F220C	C/2F
WT 1		0.4	0.1	0.04	0.19	0.0014	< 0.0001	0.0012
WT 2			0.02	0.0045	0.33	0.0009	< 0.0001	0.0005
2F/A 1				0.6	0.02	0.0002	< 0.0001	0.0002
2F/A 2					0.01	0.0001	< 0.0001	0.0001
F220A						0.0526	0.0015	0.0855
3F/A							0.0335	0.63
F220C								0.0065
C/2F								

Table S3: Listing of p-values between MMGBSA binding energies of various pairs of protein systems. The p-values were calculated from comparison of the mean and standard deviation values of the MMGBSA energies for each construct (see also Figure 6 in the main text). The abbreviations of the opsin constructs used in this table are as follows: WT 1 – WT Mode 1, WT 2- WT Mode 2, 2F/A – F221A/F228A, 3F/A – F220A/F221/F228A, C/2F – F220C/F221A/F228A. As highlighted in the red color, the dimerization energy for the F220C system is significantly different from the constructs that do not contain mutations at position 220, including the wild type dimers.
